# Ultrafast visualization of crystallization and grain growth in shock-compressed SiO_2_

**DOI:** 10.1038/ncomms9191

**Published:** 2015-09-04

**Authors:** A. E. Gleason, C. A. Bolme, H. J. Lee, B. Nagler, E. Galtier, D. Milathianaki, J. Hawreliak, R. G. Kraus, J. H. Eggert, D. E. Fratanduono, G. W. Collins, R. Sandberg, W. Yang, W. L. Mao

**Affiliations:** 1 Shock and Detonation Physics, Los Alamos National Laboratory, PO Box 1663, Los Alamos, New Mexico 87545, USA; 2 Stanford Institute for Materials and Energy Sciences, SLAC National Accelerator Laboratory, 2575 Sand Hill Road, Menlo Park, California 94025, USA; 3 Linac Coherent Light Source, SLAC National Accelerator Laboratory, 2575 Sand Hill Road, Menlo Park, California 94025, USA; 4 Institute for Shock Physics, Washington State University, PO Box 642816, Pullman, Washington 99164, USA; 5 Shock Physics, Lawrence Livermore National Laboratory, 7000 East Avenue, Livermore, California 94550, USA; 6 Center for Integrated Nanotechnologies, Los Alamos National Laboratory, Los Alamos, New Mexico 87545, USA; 7 HPSynC, Carnegie Institution of Washington, 9700 South Cass Avenue, Argonne, Illinois 60439, USA; 8 Center for High Pressure Science and Technology Advanced Research, 1690 Cailun Road, Shanghai 201203, China; 9 Geological Sciences, Stanford University, 367 Panama Street, Stanford, California 94305, USA

## Abstract

Pressure- and temperature-induced phase transitions have been studied for more than a century but very little is known about the non-equilibrium processes by which the atoms rearrange. Shock compression generates a nearly instantaneous propagating high-pressure/temperature condition while *in situ* X-ray diffraction (XRD) probes the time-dependent atomic arrangement. Here we present *in situ* pump–probe XRD measurements on shock-compressed fused silica, revealing an amorphous to crystalline high-pressure stishovite phase transition. Using the size broadening of the diffraction peaks, the growth of nanocrystalline stishovite grains is resolved on the nanosecond timescale just after shock compression. At applied pressures above 18 GPa the nuclueation of stishovite appears to be kinetically limited to 1.4±0.4 ns. The functional form of this grain growth suggests homogeneous nucleation and attachment as the growth mechanism. These are the first observations of crystalline grain growth in the shock front between low- and high-pressure states via XRD.

## Abstract (web summary)

Pressure- and temperature-induced phase transitions have long been studied, but little is known about the processes by which the atoms rearrange. Here, the authors present *in situ* measurements on shock compressed fused silica, revealing an amorphous to crystalline high pressure stishovite phase transition.

## Graphical abstract

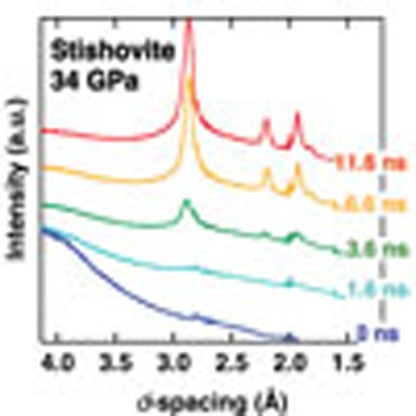

Predicting the atomistic structure of materials under conditions of extreme pressure and temperature^1^ using state-of-the-art simulation capabilities currently cannot address the timescale and mechanistic pathway of material phase transitions. Understanding the time dependence of material phase transitions has been a continued area of scientific research since the observation of shock wave propagation associated with the α–ɛ phase transition in iron^2^,^3^. A shock wave, the fastest mechanical loading that can be applied to a material, provides a nearly instantaneous change in thermodynamic conditions from which material-based dynamics controlling transitions between state or physical properties, can be measured. At the macroscopic level, Dolan *et al*.^4^ demonstrated the complexity and time dependence of a material phase transition by measuring changes in the index of refraction via optical imaging of shock-compressed water. However, obtaining atomistic data in the non-equilibrium state during the process of a material phase transition has remained elusive until now.

To understand the fundamental physics that govern atomic interactions, measurements are required at the relevant timescale and length scale. X-ray diffraction (XRD) has been used for over a century to study the atomic structure and more recently the structural changes associated with the application of pressure and temperature. The application of XRD to shock-induced phase transitions began with Johnson and Mitchel^5^ on single crystals, demonstrating the ability to measure structural changes during dynamic compression. More recently, studies on dielectrics (for example, refs 6, 7) and semiconductors or metals (for example, refs 8, 9, 10, 11, 12) have observed atomic structural transitions via nanosecond time-resolved XRD, in some cases constraining the atomic pathways, for crystalline–crystalline phase transitions. Recent *in situ* XRD experiments using short-pulse X-ray probes combined with laser-induced shocks^13^ investigated the atomistic strain time dependence associated with stress-induced plastic relaxation processes—a process too rapid to be diagnosed by other means^14^. Ongoing work using *in situ* XRD measurements has contributed to our understanding and interpretation of continuum wave profile measurements of shock-induced phase transitions^15^,^16^.

Using the high-brightness short-pulse Linac Coherent Light Source (LCLS) X-ray free-electron laser (XFEL) we report the first results of shock-induced nanosecond nucleation and growth of a high-pressure crystalline phase from initially amorphous material. Debye–Scherrer patterns are recorded during the transit of a shock wave through fused silica (SiO_2_). Temporally resolved XRD patterns clearly demonstrate the growth of crystalline stishovite out of the original amorphous fused silica. We measure the diffraction peaks widths that provide information on the size, and hence rate of growth of the nano-crystallites that form. Though a disorder to ordering process is expected to be slow, we find that the stishovite grains nucleate and grow rapidly, within the first few nanoseconds, and the growth trend supports a coalescence growth model rather than a diffusion-based mechanism. These data and present analysis are the first demonstration of shock-induced crystallization of an amorphous material via femtosecond diffraction and will lead to a greater understanding of important problems in shock physics and their relation to geophysics.

## Results

### *In situ* XRD

**Figure 1:**
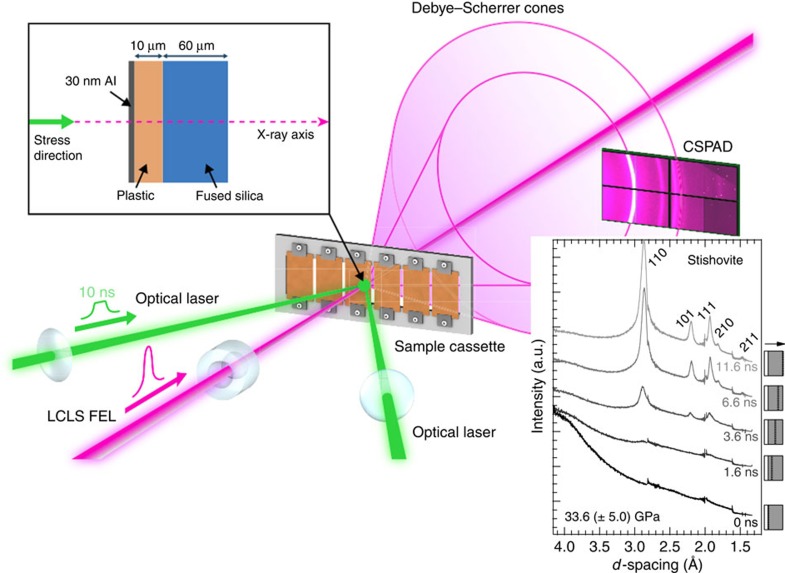
Experimental configuration of the XFEL probe and optical laser. The lattice response of the sample was captured in a Debye–Scherrer geometry. Inset: example of XRD resulting from azimuthal integration of CSPAD data for a suite of time delays under shock compression. A schematic of the target is shown on the right side for each time delay (white: plastic; grey: fused silica). A dashed line indicates the approximate location of the shock front; arrow is the shock propagation direction.

Atomic structure measurements of uniaxially shock-compressed fused silica were made using transmission *in situ* XRD with 8 keV X-rays from the XFEL at the Matter in Extreme Conditions end-station of the LCLS (Fig. 1). XRD from each pump–probe experiment, recorded on the Cornell-SLAC Pixel Array Detectors (CSPADs) (Supplementary Figure 1), is azimuthally integrated as a function of X-ray scattering angle (2*θ*) (see Methods). The applied pressure *P* from laser ablation was determined using the known fused silica principal Hugoniot^17^ and shock speed (Supplementary Methods, VISAR Analysis Details). Applied pressures of 33.6±5.0, 18.9±3.0, 7.6±1.2 and 4.7±0.8 GPa were set by the incident laser intensity. XRD measurements are spatially integrated over the whole sample and therefore the diffraction measures varying contributions from ambient and compressed SiO_2_ as a function of time due to the shock wave propagation. Time zero is defined as the time when the shock wave enters the SiO_2_.

### Diffraction observations

A first sharp diffraction peak (FSDP) from fused silica (starting density, 2.20 g cm^−3^) is centred at 2*θ*∼21.6°, consistent with previous work^18^ at ambient conditions. The observed intensity of this FSDP decreases with increasing pump–probe delay time. At the lower applied pressures the FSDP shifts to smaller *d*-spacing, indicative of a compressed amorphous material, before the phase transition initiates. The high-pressure phase is observed by the formation of azimuthally (about the X-ray beam axis) symmetric crystalline diffraction rings that are indexed as octahedrally coordinated high-pressure crystalline stishovite (tetragonal, *P*4_2_/mnm). The relative intensities of the first four Bragg reflections (110), (101), (111) and (210) and azimuthal symmetry show no preferred orientation (that is, intensities are comparable to powder diffraction^19^). Details of Rietveld refinements of example diffraction patterns are shown in Supplementary Figure 2 and Supplementary Methods: Rietveld Refinements.

**Figure 2:**
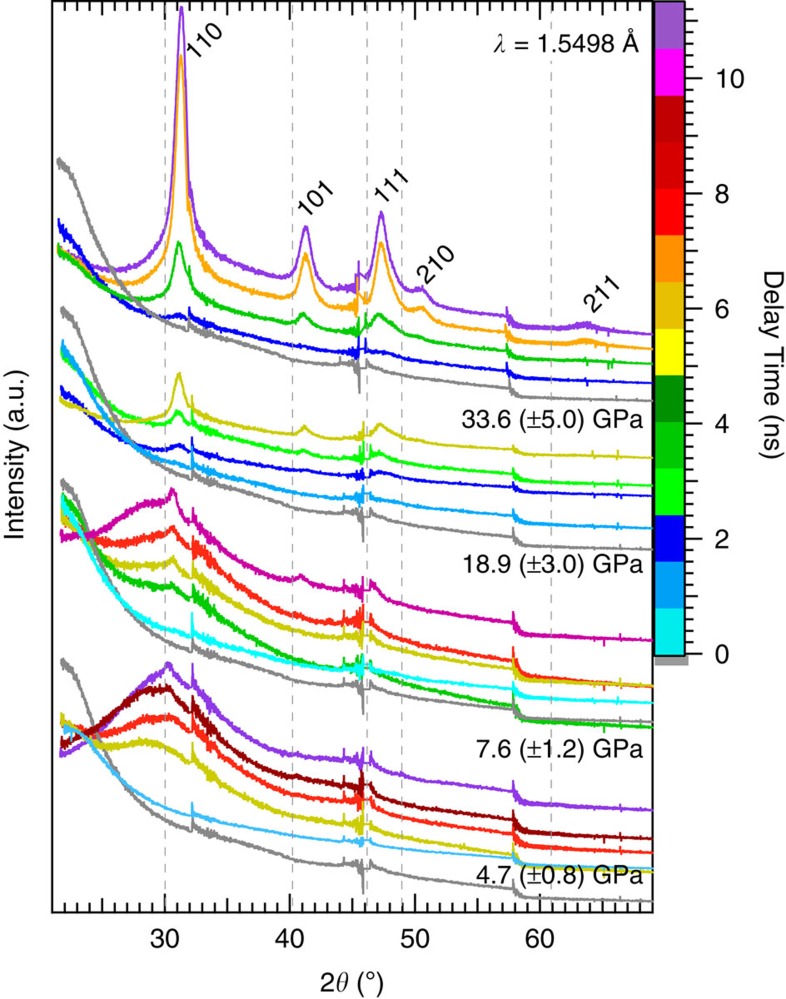
Multiplot of XRD data. Stishovite peaks are labelled at the top; ambient condition positions (grey dashed lines). Traces are clustered according to applied pressure where each colour indicates a different delay time (grey is X-ray only). Offset along the *y* axis is arbitrary for viewing clarity. Discontinuities in the traces are seen at 32.5°, 46.0° and 58.0° 2*θ* due to spacing between the application-specific integrated circuits of the CSPADs.

At 33.6 GPa time-resolved snapshots show the structural changes (Fig. 1) during the reconstructive transformation to stishovite (density measurement, 4.61(7) g cm^−3^, is consistent with previous work^20^). By 3.6 ns, the data show the clear emergence of crystalline stishovite peaks. With increasing probe delay time, the intensity of these diffracted peaks increases as the shock front moves further into the sample and the XFEL beam probes a larger volume fraction of the high-pressure stishovite phase. Figure 2 shows the integrated diffraction data for each applied pressure and delay time.

XRD patterns at 4.7 and 7.6 GPa show a super-positioning of a very broad peak centred at 2*θ*=30.4° (±0.3) and the emergence of a narrow peak fitting the (110) position of stishovite at later time delays. We interpret this as observing first the compression of the amorphous silica, as documented by a shift in the FSDP to smaller *d*-spacing, followed by the onset of stishovite by 3.7–9.3 ns delay times. The appearance of stishovite at the lower applied pressure is within the stability field of coesite as determined from static compression work (Supplementary Methods: Diffraction Interpretation Details).

### Peak width analysis

**Figure 3:**
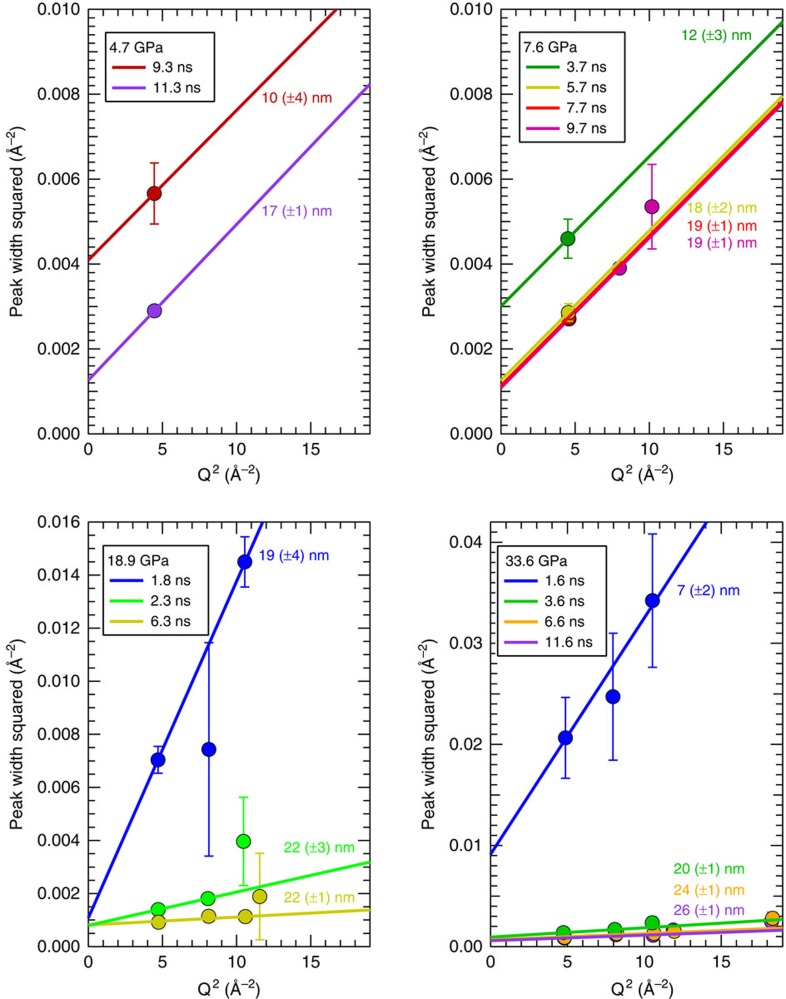
Analysis of peak widths versus lattice plane position at four applied pressures. *Q* is related to the 2*θ* peak position by: 
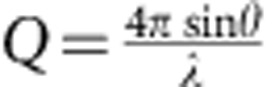
; X-ray wavelength *λ*=1.5498 Å. Dots are the measured points and the lines are the weighted linear fits. Colour code is the same as in Fig. 2. The lack of more than one Bragg peak at a given time delay for the 4.7 and 7.6 GPa data limits our ability to directly measure the slope. Therefore, the *A* parameter determined from the 7.6-GPa, 9.7-ns shot (yielding a r.m.s. strain of 10^−3^) is used for all other 4.7- and 7.6-GPa traces, providing an upper bound on the grain size. For the 18.9- and 33.6-GPa shots *ɛ* decreases with increasing delay time from 10^−3^ to 10^−4^.

The geometry-related peak broadening of the Debye–Scherrer cones projected on the CSPADs is corrected and the instrumental broadening measured using a CeO_2_ powder standard (Supplementary Fig. 2 and Supplementary Methods: Rietveld Refinements). Peak widths of the stishovite are markedly broader than the standard. The extra broadening is interpreted as coming from a combination of size and strain broadening. The average grain size and root mean squared (r.m.s.) strain distribution can be extracted from the data by using a modified Warren–Averbach^21^ analysis. For our analysis, a symmetric strain profile is assumed and each peak is fit with a Gaussian distribution: 
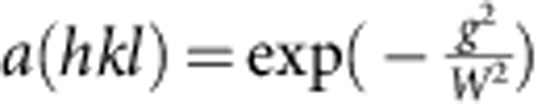
, where *g* is the scattering vectors centred at lattice plane (*hkl*), whose inverse plane spacing (*Q*_*hkl*_) and peak width *W* is related by: *W*^2^=*B*^2^+*A*^2^*Q*_*hkl*_^2^ (Fig. 3). Peak width parameters *A* and *B* are related to strain and grain size, repectively^11^. A weighted linear fit to *W*^2^ plotted as a function of *Q*_*hkl*_^2^ provides the value for the *y* intercept: 
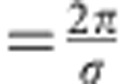
, where *σ* is an average grain size, and the slope: 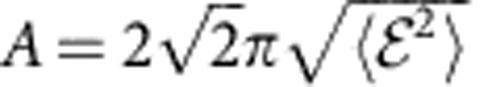, where 
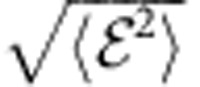
 is the r.m.s. strain assuming a Gaussian strain distribution within the grains probed (Supplementary Methods: Grain Size Determination). At early times we see 1 (±0.5) % r.m.s. variations in strain reducing at later times to 0.1 (±0.06) % r.m.s.

## Discussion

**Figure 4:**
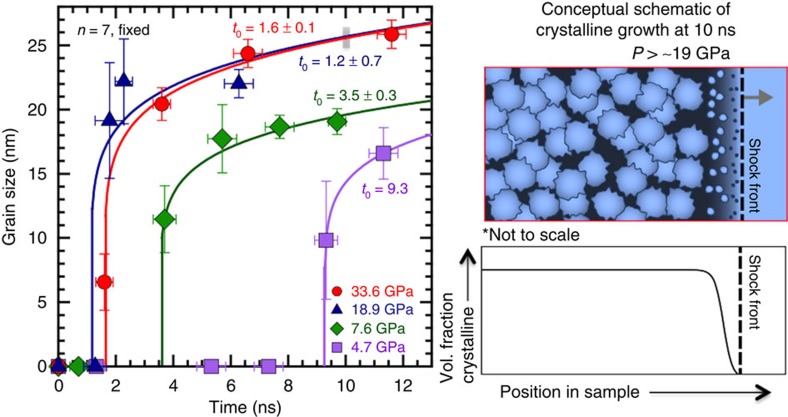
Experimentally determined average grain sizes as a function of delay time. Different colours/symbols are for SiO_2_ at different applied pressures. Fits are from a simple growth model. Cartoon on the right (for 33.6 GPa at 10-ns delay, grey box) illustrates our interpretation of grain growth behind the shock front (black dashed line, propagation direction is grey arrow) showing a distribution of grain size increasing with distance from the shock front. A qualitative trend for probable grain density^23^ as function of time (or distance) is also shown.

Tabulated average grain sizes plotted as a function of probe times (Fig. 4) show the nucleation and growth of the high-pressure phase. Similar to other nanocrystalline growth^22^ the evolution of the mean particle size versus time resembles the trend predicted by classical growth models^23^ where the size of the particles, *D*, is proportional to growth time *t*. A description similar to Huang *et al*.^24^ is used: *D*=*k*(*t−t*_0_)^1*/n*^ where *k*(*T*) is a temperature (*T*)-dependent material constant appropriate to exponent *n*, and *t*_0_(*P*) is nucleation time. *n* is associated with transformation and growth mechanisms^24^,^25^,^26^, that is, when *n*≤4 growth is diffusion related, however, if *n*>4, growth could be described by attachment or coalescence events. The data were fit with a single best-fit value of *n*=7 for all applied pressures, indicating perhaps a coalescence grain growth regime due to homogenous nucleation^27^ is most appropriate for the stishovite. *k*(*T*) and *t*_0_(*P*) are expected to vary with applied shock pressure by the change in thermodynamic state. The *t*_0_ decreases and *k*(*T*) parameter increases with increasing applied pressure (and therefore increasing temperature). There is little difference in the observed nucleation time (1.4 ns), growth rate or plateau for 18.9 and 33.6 GPa data, suggesting that the higher applied pressure shots are kinetically limited in the nucleation and growth of stishovite (for fitting details see Supplementary Methods: Grain Growth Model).

Our results clearly show the majority of the sample becomes stishovite during shock compression. Compared with temperature-only driven studies (for example, ref. 28) looking at nucleation and growth of crystallites from amorphous starting samples, our experiments probed shorter length- and timescales, where we see order of magnitude faster growth and smaller grains. In fact, the timescale for stishovite nucleation is surprisingly fast, in particular at the higher applied stresses, it is markedly faster than what is expected for a diffusion-mediated process^27^. Other diffusionless transformations (that is, martensitic transitions in iron^16^,^27^) take place in a few nanoseconds or less. Reconstructive transitions are thought to be diffusive in nature^5^, however we have shown the mechanism of transformation may be better suited by coalescence events in a homogeneous nucleation regime.

Stishovite is found at bolide-impact craters on the Earth's surface presumably generated by a shock wave process. And contrary to some studies concluding that a dense amorphous phase, rather than crystalline stishovite forms along the SiO_2_ Hugoniot^29^, we have shown on the laboratory timescale, we directly observe formation and growth of stishovite. Shock recovery experiments only find a trace amount of stishovite^30^ but the material goes through a release pathway. Therefore, our new data on stishovite forming on compression may constrain the formation of the diaplectic (sixfold coordinated) glass and coesite to the release path—an important clue to unravelling the impact history of Earth and the solar system.

## Methods

### Experimental setup

Using the Matter in Extreme Condtions instrument at the LCLS^31^, quasi-monochromatic (d*E*/*E*=0.2–0.5%), fully transverse coherent, 7.952(30) keV X-ray pulses of 60-fs duration with an average of ∼10^12^ photons per pulse, were incident over a 75-μm diameter spot on the target package. Wafers of amorphous Nikon synthetic fused silica (SiO_2_) prepared by melt quenching were double-side parallel polished to a thickness of 60 μm and diced into 2 × 2 mm individual targets. The wafers include tens of parts per million (p.p.m.) values of OH and Cl with <1 p.p.m. for other cations. These targets were batch coated with 10 μm of plastic (glow discharge polymer deposition of trans-2-butene, 1C:1.3H (ref. 32)) to serve as the ablator. An X-ray-only shot was collected before each drive shot as a reference. The 75-μm XFEL beam spot did not produce any observable X-ray damage to the target. Using phase plates on the optical drive laser, a 200-μm diameter flat-top laser spot was used to achieve focal spot intensity of ∼10^12^ W cm^−2^. The angle between drive laser arms and XFEL probe is 6°. An ablation-driven compression wave was launched parallel to the sample normal over a 10-ns quasi-square pulse profile from a frequency-doubled Nd:Glass laser system (*λ*=527 nm). The optical laser and X-ray beam were spatially overlapped and operated in single-shot mode. The absolute time zero corresponds to overlap of their leading edges. For each shot, a time delay was selected for the XFEL pulse relative to the optical laser pulse with a jitter of 0.3–0.5 ns (which is displayed as the temporal uncertainty for Fig. 4). This delay time was verified by oscilloscope traces captured for each shot. For the purposes of discussing the kinetics in the SiO_2_ only, we establish a relative time zero defined as the time at which the pressure wave reaches the interface between the plastic ablator and the SiO_2_. The transit time through the plastic ablator varies as a function of drive energy and was determined from VISAR measurements (see Supplementary Methods: VISAR Analysis Details). The combined use of a pressure–irradiance scaling and the transit time provides constraints on the applied pressure for each shot. The pump–probe delay scans at several nanosecond intervals enabled collection of a time series of XRD patterns in transmission geometry. XRD patterns were captured by CSPADs constructed of individual application-specific integrated circuits^33^. Maximum azimuthal angle coverage was 23°. One target was shot per time delay selected.

### Diffraction normalization

Background subtraction and normalization of each trace to volume shocked was explored. First, a dark pattern without X-rays was subtracted from every trace (examples of darks and ambient condition SiO_2_ traces given in Supplementary Fig. 3). *I* is the normalized intensity (equal to the integrated intensity of the entire sample) with a contribution from each region of the target: a (ablator), u (unshocked SiO_2_) and s (shocked SiO_2_). We found the signal from the plastic ablator (*I*_a_) only contributed ∼25 counts on the CSPADs, and is therefore taken to be negligible in the normalization calculations, therefore, *I*_bkgd_=*I*_u_ is determined from the X-ray-only pre-shot trace. Defining *n*_u_ as the fraction of SiO_2_ unshocked and *n*_s_=1−*n*_u_ as the fraction of SiO_2_ shocked, the signal from a shot (*I*_sig_) is defined as *I*_sig_=*I*_u_*n*_u_+*I*_s_*n*_s_. Therefore, the normalization factor (*I*_sig_−*n*_u_*I*_bkgd_)/*n*_s_, applied to every trace in Fig. 2 gives Supplementary Fig. 4. However, due to uncertainties in the contribution of the background shot to shot we cannot accurately determine the phase fraction of the individual components, that is, uncompressed amorphous, compressed amorphous or stishovite, and therefore only estimate the relative intensities of the uncompressed region to compressed region.

## Additional information

**How to cite this article:** Gleason, A. E. *et al*. Ultrafast visualization of crystallization and grain growth in shock-compressed SiO_2_. *Nat. Commun.* 6:8191 doi: 10.1038/ncomms9191 (2015).

## Supplementary Material

Supplementary Information Supplementary Figures 1-4, Supplementary Table 1, Supplementary Methods and Supplementary References
