# Ultrafast visualization of crystallization and grain growth in shock-compressed SiO_2_

**DOI:** 10.1038/ncomms9709

**Published:** 2015-10-13

**Authors:** A. E. Gleason, C. A. Bolme, H. J. Lee, B. Nagler, E. Galtier, D. Milathianaki, J. Hawreliak, R. G. Kraus, J. H. Eggert, D. E. Fratanduono, G. W. Collins, R. Sandberg, W. Yang, W. L. Mao

Nature Communications 6: Article number: 8191 doi: 10.1038/ncomms9191 (2015); Published 09042015; Updated 10132015

An incorrect version of the Supplementary Information was inadvertently published with this Article and did not show axes labels for Supplementary Figs 2–4. The HTML has now been updated to include the correct version of the Supplementary Information.

## 

## Supplementary Material

Supplementary Information Supplementary Figure 1-4, Supplementary Table 1, Supplementary Methods and Supplementary References

