# Supplementary material for: Ultrafast visualization of crystallization and grain growth in shock-compressed SiO2
Source: Nat Commun. 2015 Oct 13;6:8709. doi: 10.1038/ncomms9709 (PMC4633954; doi:10.1038/ncomms9709)
Supplement: Supplementary file 1 [file ncomms9709-s1.pdf]

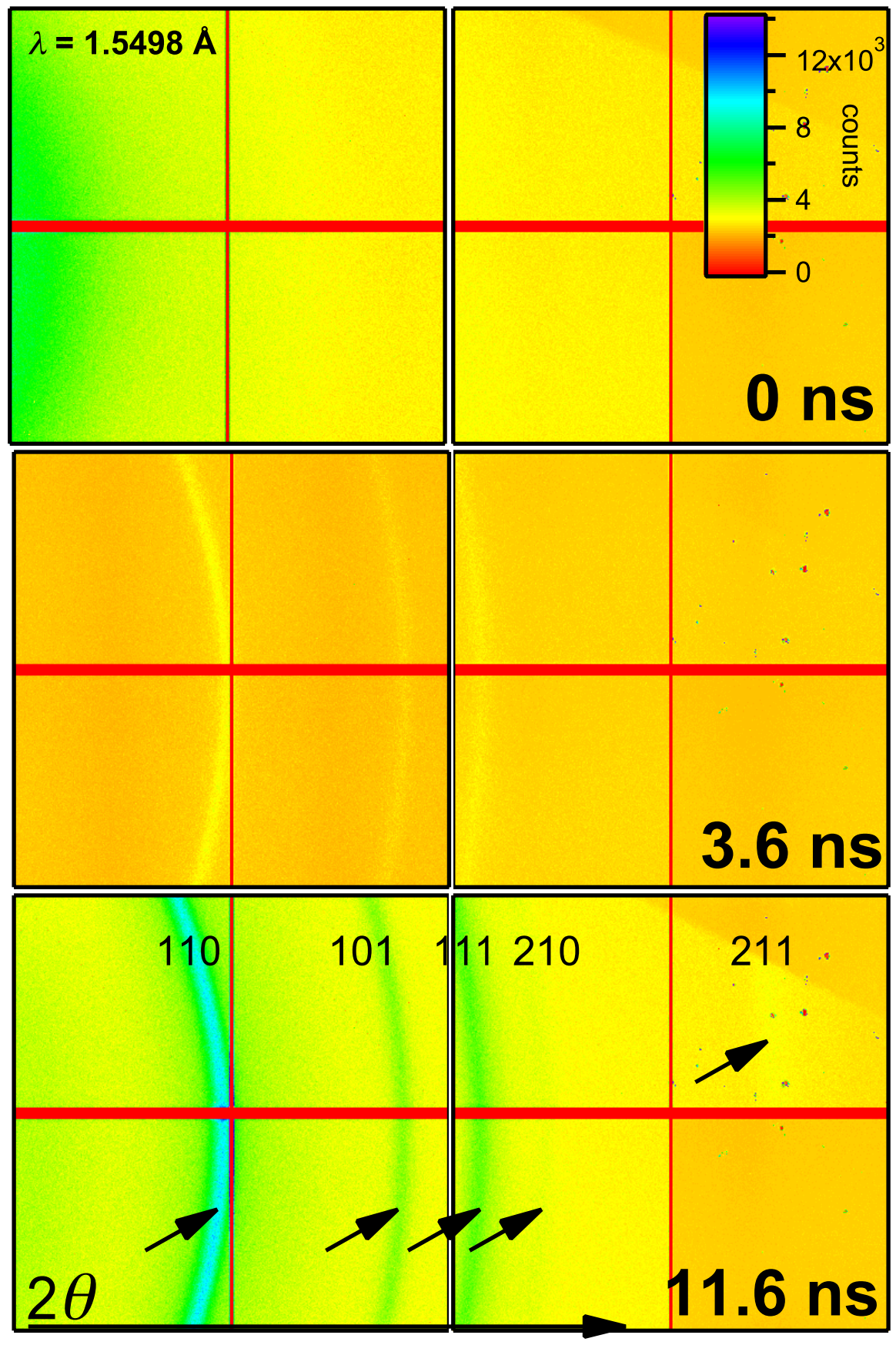

Supplementary Figure 1: **Raw CSPAD data.** Example raw images are displayed for an X-ray only pattern and two time delays at 33.6 GPa to provide a sense of the data quality without darks or background subtraction. Color scaling has been matched for all CSPAD ASICs at maximum 14,000 counts. By 11.6 ns we see 5 peaks indexed as pure stishovite.

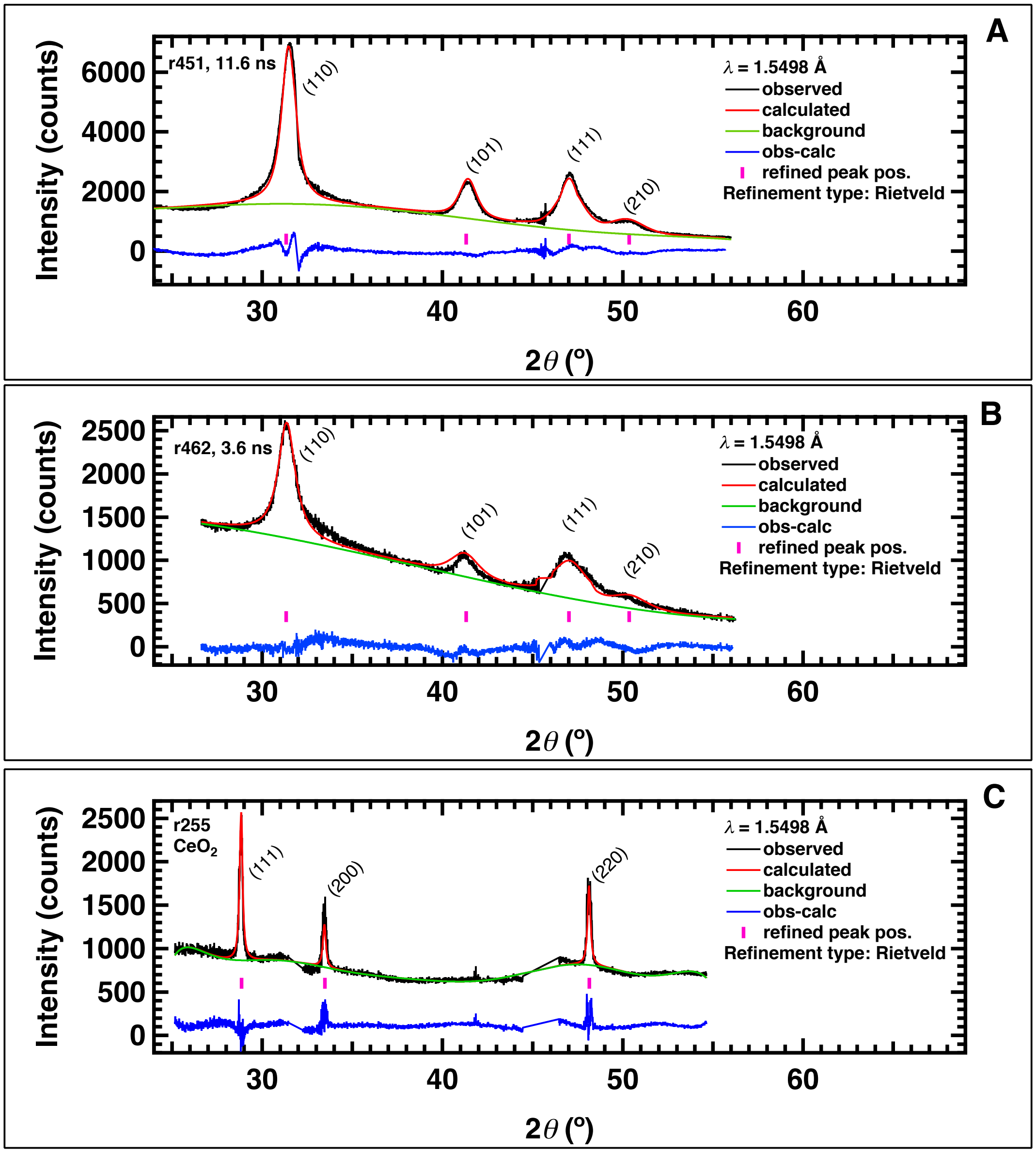

Supplementary Figure 2: **Diffraction refinements**. General Structure Analysis System (GSAS) software[1] with EXPGUI[2] were used to perform Rietveld refinements on integrated sample diffraction data (two examples at different time delays from the 33.6 GPa shots are shown here as panels (A) and (B)) and on CeO_2 standard (panel (C)). Pixels associated with the spaces in between ASICs of the CSPADs have been removed for refinement.

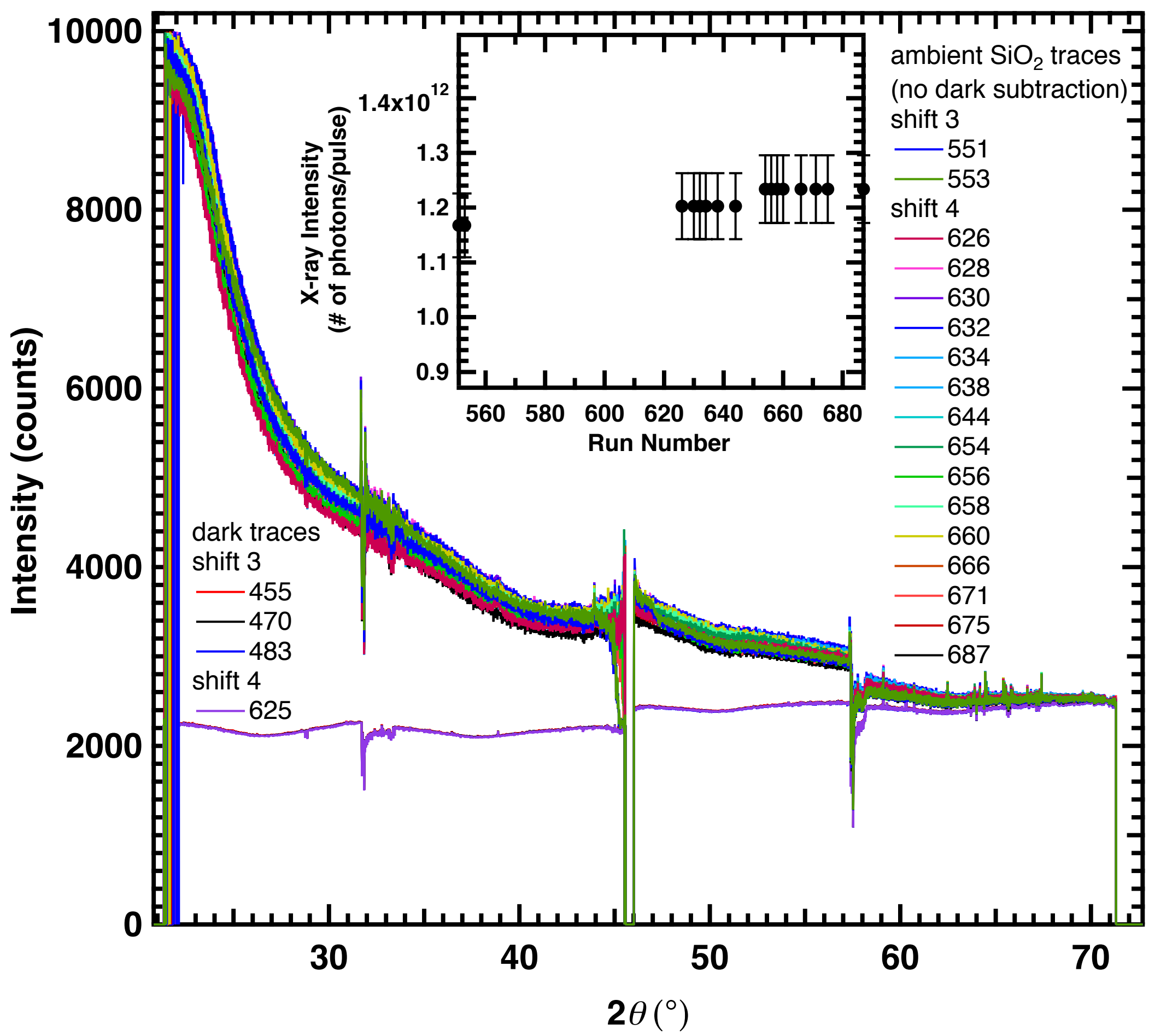

Supplementary Figure 3: **Example traces from ambient condition fused silica.** These show consistency in sample intensity over several runs and across 12-hour shifts (e.g., 3 and 4). Examples of dark patterns are also shown to give ~2000 counts. Note that some ASICs show an apparent, artificial offset in intensity for the dark patterns (e.g., 45-57°). *Inset:* Graph of incident X-ray photon energy as a function of runs listed for the X-ray only fused silica patterns. On average, 10^{12} photons per pulse per shot were recorded for this experiment.

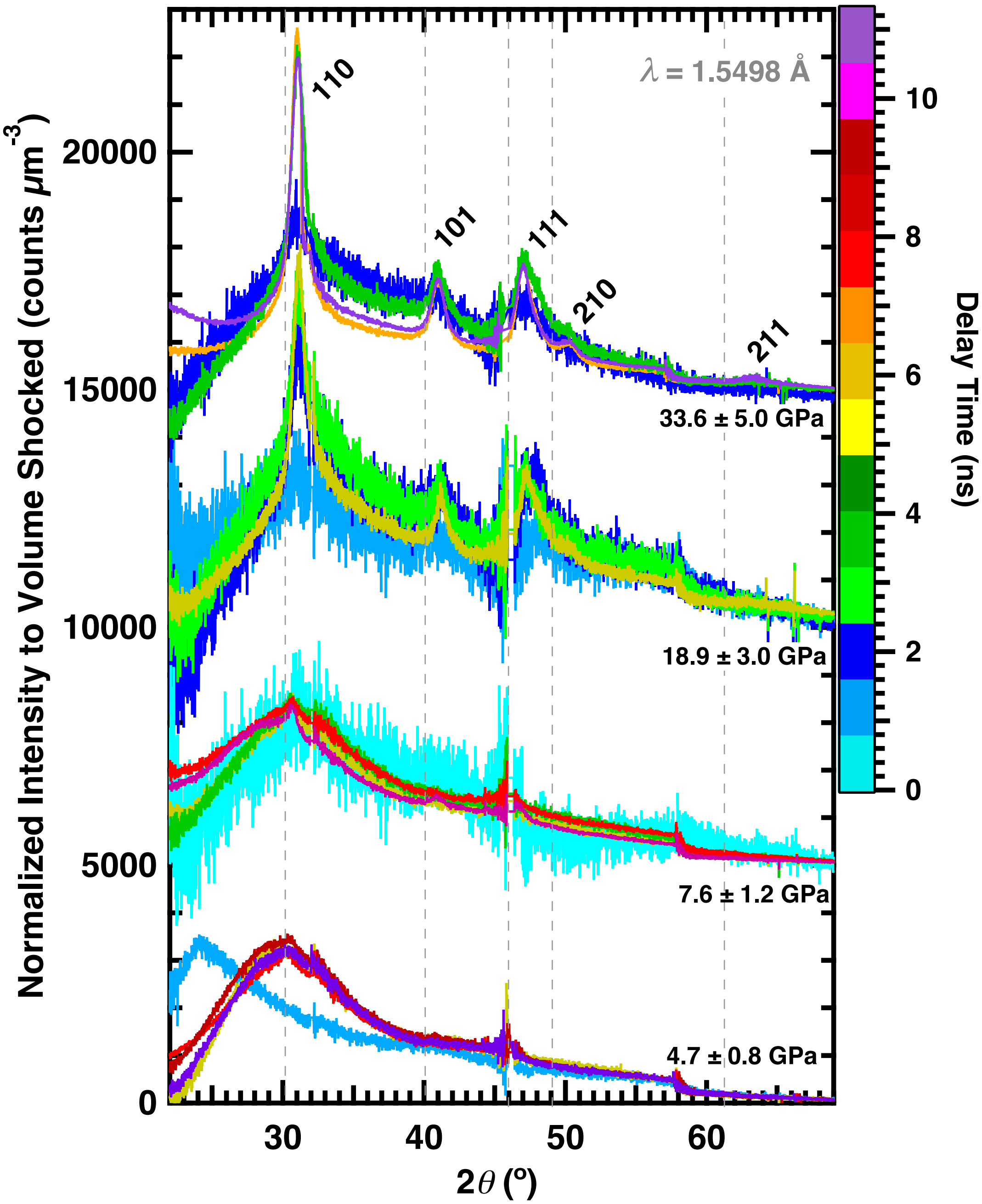

Supplementary Figure 4**: Volume shocked normalized data.** These data are the traces from Fig. 2 (same color code), following the normalization prescription outlined in the Methods section. Traces for 7.6, 18.9, and 33.6 GPa are offset vertically (identical offset applied to each pressure grouping) for visual clarity.

Supplementary Table 1: **Unit Cell and Fit Parameters for Stishovite and Ceria**

**Unit Cell and Fit parameters**					
Stishovite					
	**Run**	**Volume (Å^3)**	***a*** **(Å)**	***c*** **(Å)**	**reduced-χ^2**
	r451	42.89(1)	4.0458(8)	2.6200(8)	2.26
	r462	43.15(2)	4.0577(6)	2.621(1)	1.95
CeO_2					
	r255	158.09(1)	5.4072(1)	n/a	1.04

Supplementary Table 1: The lattice parameters (a,c), unit cell volumes (V) and goodness-of-fit factors (reduced-χ^2) extracted from Rietveld refinements of two example traces of stishovite and XRD calibrant, Ceria (CeO_2).

Supplementary Methods

VISAR Analysis Details

Velocity Interferometer System for Any Reflector (VISAR)[3] data was collected simultaneously for each drive intensity and XFEL delay combination. The applied pressures listed in this paper are determined using the transit time through the sample package to calibrate a pressure-irradiance scaling law for the GDP ablator, and then using the scaling law exponent provided in Swift and Kraus[4] to determine the sample pressure for the lower energy shots. This method is preferred to using the transit times at the lower stress states to directly determine the shock pressure from an inferred wave velocity because of the complicated compressive response of fused silica below ~25 GPa. The variation in strain for the material involved in this style of compression is significant, however, the volume fraction is less than 10%.

To determine the transit time through the entire sample, the drive laser was accurately co-timed with the streak cameras used for the VISAR diagnostic. Possibly due to break-up of the fused silica material upon breakout of the shock-wave at the free surface, it was impossible to determine the free surface velocity of the fused silica. Instead, we measure a total transit time through the GDP ablator and the fused silica sample.

The Hyades Radiation Hydrodynamics code[5] was used to model the wave propagation through the GDP ablator and the fused silica sample. SESAME 7592 and 7386 equations of state were used for the GDP ablator and fused silica, respectively[6]. To match the transit time and associated scatter, 11.56 ±0.33 ns, through the fused silica and ablator at the highest laser energy, 32 J, required a final shock pressure in the ablator and

fused silica of 33.6 ±3.7 GPa. This uncertainty in the shock state includes the scatter in the measured transit times as well as the uncertainty in the EOS models (~3%) and the uncertainty in the total sample thickness (~3%). To scale from the highest laser energies to the lower energy shots, 16, 5, and 3 J, we used the pressure-irradiance scaling law presented in Swift and Kraus[4]. For a 5% uncertainty in the scaling law exponent, we find that the shock states in the fused silica for 16, 5, and 3 J are 18.9 ±2.3, 7.6 ±1, and 4.7 ±0.7 GPa, respectively. These stress states assume that the shock stress was not increasing nor decreasing in stress with time. The pulse shapes were designed to produce such steady shocks, however, because we did not observe the wave profile after it has propagated through the sample, we allow for the possibility that the wave strength increases or decreases over the sample thickness by ~10%, as was deemed reasonable from our simulations[4]. Therefore, we list the applied pressures for this experiment as 33.6 ±5.0, 18.9 ±3.0, 7.6 ±1.2 and 4.7 ±0.8 GPa for drive energies of 32, 16, 5, and 3 J, respectively. It should be noted that pressure estimates using diffraction alone are within VISAR uncertainties – including a 1%, or less, temperature correction to unit cell volumes[7] due to shock heating. Admittedly, the Hugoniot stress state is only applicable to the high pressure equilibrium state, not material within the shock front or transition region. However, in lieu of a better, more direct pressure measurement in the transition region, and the need to sort the datasets according to relative stress state to make quantitative comparisons, we settle on these assignments of the applied pressures as the nomenclature for this paper.

Rietveld Refinements

Using General Structure Analysis System (GSAS) software[1] with EXPGUI[2] we perform Rietveld refinements on integrated diffraction data; two examples at different time delays from the 33.6 GPa shots are shown in Supplemental Mat. Fig. 2. The goodness-of-fit factor (reduced-χ^2) and lattice parameters (a,c) and unit cell volume (V) for these traces derived from each fit are listed in Supplementary Table 1. Thermally corrected pressures derived from our fitting (using our unit cell volumes and the equation of state from reference Wang et al.[7]) are found to be similar to each other (as expected, considering the same drive conditions were used) and within the uncertainty of the VISAR derived stress value of 33.6 ± 5.0 GPa. No preferred orientation corrections in GSAS were required to provide a match of (110), (101), (111) and (210) peak intensities to previously published powder data found in the crystallographic information file (cif)[8]. This cif provided the starting phase information for GSAS. From this comparison to Ross et al.[8] peak intensities, we find our diffraction data lack preferred orientation. Note that the right most ASICs (at the highest 2θ) for each time slice show significant damage (e.g., not recording any intensities at all, damaged pixels, shadowing). Therefore, only peak position is recorded for (211) due to difficulties extracting reliable relative intensities in these quadrants. It should be noted that for our refinements, no amorphous component was considered when fitting the background. Since the goal was to refine the crystalline peak position and intensity, we decided not to include the diffuse component in the refinement fitting and the background was fit with a Chebyshev polynomial in GSAS irrespective of the location of the shock front in the sample and percent shocked or unshocked material. We also note that the largest contribution to the reduced-χ^2 value is

derived from the (110) peak straddling the edge of ASICs such that if we artificially fill in those intensities the reduced-χ^2 value decreases significantly.

To calibrate our sample-to-detector distances, detector tilts and determine instrumental broadening we used a NIST Standard Reference Material (SRM) 674b CeO_2 powder. A Rietveld refinement (Supplementary, Fig. 2) of CeO_2 powder shows much narrower peaks than for the stishovite grown during shock compression. From our Rietveld fits, finding GSAS Gaussian parameters GU = 0.5, GV = 0.0, GW = 0.0 and the "Caglioti" function $FWHM^2 = U \tan^2\theta + V\tan\theta + W$, (Ref. 9) we can monitor the instrumental broadening as a function of diffraction angle. The peak width of the highest 2θ stishovite peak, (210), included in the peak broadening analysis for grain size determination, is five times broader than the instrumental FWHM.

Diffraction Interpretation Details

We find the compressed fused silica peak is centered at ~2.10(5) $Å^{-1}$. If we assume 4-fold coordination for this glass (i.e., comparing to work from Sato and Funamori[10] for a low density amorphous (LDA) phase), the pressure estimate is 19.7 (+/- 1.4) GPa. This contrasts the (110) peak position of stishovite and VISAR data (in combination with the pressure-irradiance scaling) suggesting a much lower applied pressure of 4.7(8) GPa. We speculate the glass component is transitioning from a 4-fold to 6-fold coordination and a strict comparison to a pressure derived from only a 4-fold glass structure would be unfair and manifest as an inconsistency. In other words, a density or pressure comparison would only be valid if we: a) knew the structure of our glass phase, and b) found that structure to be comparable to the findings of Sato and

Funamori[10]. However, given the 2θ coverage of our data, we cannot assess the glass structure and therefore a pressure comparison is not appropriate. Furthermore, the stress conditions we explore in this experiment are within the "mixed phase region" of fused silica which makes interpretation of these data complex and in no way are we indicating these findings are definitive, rather a starting place for exploring new physics. In an effort to provide a metric for identification (or classification) of behaviors as a function of pressure we decide to use the crystalline diffraction/VISAR applied pressure value of 4.7 GPa for the rest of the paper.

The development of stishovite peaks at longitudinal stresses of 4.7 and 7.6 GPa is contradictory to the SiO_2 *P-T* phase diagram as determined from static compression experiments[11]. In our experiments, diffraction peaks matching coesite are never seen. To the best of our knowledge, coesite has not been produced or recovered from shock wave experiments[12] due to the quenching path necessary to form metastable coesite[13]. We explain this by the sluggish nature of the transition quartz to coestite and the over-driven pressure conditions on a reduced timescale allowing the higher symmetry, metastable stishovite phase to form first. In other words, if the transition from fused silica is kinetically inhibited from going to coesite then it may be thermodynamically consistent to transition to stishovite in the coesite stability region. A similar phenomenon has been observed in water-ice. Metastable ice VII forms from the liquid in the ice VI stability regime and metastable ice VII forms from the high density amorphous phase in the ice VI stability regime[14,15]. Phases of simpler symmetry can be seen in the stability regime of less symmetric phases and indicates a kinetic-control process influenced by a low interface free energy.

Grain Size Determination

We cross-check our estimate of grain size against the commonly used Scherrer Equation which does not contain a strain broadening term[16]: $\sigma = \dfrac{K\lambda}{\beta\cos\theta}$, where σ = grain size; K = dimensionless shape factor (commonly set to 0.9); λ = X-ray wavelength; β = line broadening at full width at half maximum (FWHM) minus instrumental broadening (0.03°); θ = Bragg angle. Since this equation is suited for a single Bragg peak, after calculating the grain size for each peak in a given trace, we average these values together to determine a single grain size per trace. The uncertainty extracted from each fit is also averaged to determine a total Scherrer uncertainty for the trace. As expected, we find remarkable agreement (grain size values are identical within uncertainties) compared to the Warren-Averbach[17] method using the prescription of Hawreliak et al.[18]. However, we note that the Warren-Averbach[17] method does not discriminate between inter-grain and intra-grain strain broadening.

Grain Growth Mode

In order to assume the simplest model possible, using the expression $D = k(t\text{-}t_0)^{1/n}$, we fix n to single value and let k and t_0 be free parameters. We tested a range of n values but found the curvature of the model trend did not match the data points well if we forced $n \leq 4$. At n = 6 or 8 the t_0 values were reasonable, but find the best fit for all datasets was at n = 7 (±1). A table of our fitting parameters is as follows:

**Table of fitting parameters, *n* = 7, fixed**		
**Stress (GPa)**	***k***	**t_0 (nm)**
33.6	18.9(5)	1.6(1)
18.9	18(1)	1.1(7)
7.6	15.0(5)	3.5(3)

4.7	14	9.3

For the 33.6 and 18.9 GPa data, the t_0 values are indistinguishable with the uncertainty, therefore we group them together to give an average nucleation time of 1.4 (4) ns. Note that for the 4.7 GPa trend, we have only 2 data points so the fitting is not well-constrained.

Supplementary References

1. Larson, A. & VonDreele, R. General Structure Analysis System (GSAS), Los Alamos National Laboratory Report, LAUR 86-748 (2000)
2. Toby, B.H. EXPGUI, a graphical user interface for GSAS. *J. Appl. Cryst.* **34**, 210-213 (2001)
3. Celliers, P., Collins, G., DaSilva, L., Gold, D. & Cauble, R. Accurate measurements of laser-driven shock trajectories with velocity interferometry. *Appl. Phys. Lett.* **73,** 1320-1322 (1998)
4. Swift, D. & Kraus, R. Properties of plastic ablators in laser-driven material dynamics experiments. *Phys. Rev. E* **77**, 066402 (2008)
5. Larsen, J. & Lane, S. Hyades—a plasma hydrodynamics code for dense plasma studies. *J. Quant. Spectrosc. Radiat. Transfer* **51**, 179-186 (1994)
6. Holian, K. ed. SESAME database: Los Alamos Nat. Lab. LA-10160-MS (1984)
7. Wang, F., Tange, Y., Irifune, T. & Funakoshi, K.-i. P-V-T equation of state of stishovite up to mid-lower mantle conditions. *J. Geophys. Res.* **117,** B06209 (2012)
8. Ross, N., Shu, J.F., Hazen, R. & Gasparik, T. High pressure crystal chemistry of stishovite. *Am. Min.* **75,** 739-747 (1990)
9. Kaduk, J. & Reid, J. Typical values of Rietveld instrument profile coefficients. *Powder Diffraction* **26,** 88-93 (2011)
10. Sato, T. & Funamori, N. High-pressure structural transformation of SiO2 glass up to 100 GPa. *Phys. Rev. B* **82**, 184102 (2010)
11. Boyd, F. and England, J. The quartz-coesite transition. *J. Geophys. Res.* **65,** 749-756 (1960)
12. Davies, G. Equations of state and phase equilibria of stishovite and a coesitelike phase from shock-wave and other data. *J. Geophys. Res.* **77,** 4920-4933 (1972)
13. Wackerle, J. Shockwave compression of quartz. *J. Appl. Phys.* **33,** 922-937 (1962)
14. Lee, G., Evans, W. & Yoo, C.-S. Crystallization of water in dynamic diamond-anvil cell: Evidence for ice VII-like local order in supercompressed water. *Phys. Rev. B* **74,** 134122 (2006)
15. Chen, J.-Y. & Yoo, C.-S. High density amorphous ice at room temperature. *Proc. Nat. Acad. Sci.* **108,** 7685-7688 (2011)
16. Langford, J. & Wilson, A. Scherrer after sixty years: a survey and some new results in the determination of crystallite size. *J. Appl. Cryst.* **11,** 102-113 (1978)
17. Warren, B. & Averbach, B. The effect of cold-work distortion on x-ray patterns. *J. Appl. Phys*. **21,** 595-599 (1950)

18. Hawreliak, J., Kalantar, D., Stolken, J., Remington, B., Lorenzana, H. & Wark, J. High-pressure nanocystalline structure of a shock-compressed single crystal of iron. *Phys. Rev. B* **78,** 220101 (2008)
